# Eyelid Pilomatrixomas: A Case Report and Comprehensive Literature Review

**DOI:** 10.7759/cureus.84639

**Published:** 2025-05-22

**Authors:** Georgia L Schafer, Krista Thompson, Katie Topping

**Affiliations:** 1 Ophthalmology, Loma Linda University Medical Center, Loma Linda, USA; 2 Ophthalmology, University of Texas Southwestern Medical Center, Dallas, USA; 3 Ophthalmology/Oculoplastic and Orbital Reconstructive Surgery, Naval Medical Center, San Diego, USA

**Keywords:** case report, eyelid pilomatrixoma, eyelid tumor, pathology of pilomatrixoma, pilomatrixoma, systematic literature review

## Abstract

Pilomatrixomas are rare, benign tumors of the hair follicle that can be found all over the body and, rarely, the eyelid. Despite their benign nature, pilomatrixomas can be misdiagnosed due to their similarity to other eyelid conditions. Diagnosis can be challenging and requires a combination of clinical skills, imaging studies, and histopathology. As a result, a comprehensive understanding of their presentation and management strategies is essential for healthcare providers. Herein, the unusual case of a 13-year-old female patient with a rapidly progressive eyelid pilomatrixoma is presented. A comprehensive review of the current literature regarding eyelid pilomatrixomas was performed. This literature review aims to synthesize existing research and case studies related to pilomatrixomas of the eyelid, focusing on their etiology, differential diagnosis, treatment options, and potential complications.

## Introduction

Eyelid masses are common in the pediatric population. The differential is broad and might include chalazion, dermoid cyst, vascular malformation, lipoma, fibroma, xanthogranuloma, and even malignant lesions, including rhabdomyosarcoma [1-4]. These pathologies can be difficult to differentiate clinically and often require a combination of clinical exam findings, imaging, and pathology to acquire a definitive diagnosis [5].

Pilomatrixomas are rare, benign tumors of the hair follicle that account for approximately 0.5-1% of all eyelid lesions [1,6]. These tumors are believed to occur secondary to localized mutations in hair matrix cells, and are typically secondary to spontaneous mutations [5]. Approximately 75% of cases are linked to mutations in CTNNB1, a regulator of the β-catenin/TEF-1 complex, which promotes cellular proliferation [7,8]. Unlike typical hair matrix cells, matrical cells in pilomatrixomas fail to properly form into the hair shaft [9]. Rarely, patients with multiple pilomatrixomas have a genetic syndrome such as Gardner syndrome, myotonic dystrophy, or Rubinstein-Taybi syndrome [10]. Most patients will present with a firm, mobile, and painless subcentimeter subcutaneous lesion of the head or neck. The overlying skin may be unchanged, erythematous, or blue in color [11]. Imaging with ultrasound (US) or CT shows a well-defined hyperechoic or calcific nodule [12]. Currently, there are few retrospective studies and many case reports evaluating eyelid pilomatrixomas. However, there is no comprehensive literature review and overall analysis of this pathology.

This paper includes a discussion of the case of a 13-year-old female patient with a presentation that was initially concerning for a rhabdomyosarcoma based on imaging but was ultimately confirmed to be a pilomatrixoma. It also includes a comprehensive literature review of the available literature regarding eyelid pilomatrixomas.

Despite their benign nature, pilomatrixomas can be misdiagnosed due to their similarity to other eyelid conditions. As a result, a comprehensive understanding of their clinical presentation, histopathology, and management strategies is essential for healthcare providers. This literature review aims to synthesize existing research and case studies related to pilomatrixomas of the eyelid, focusing on their etiology, diagnosis, treatment options, and potential complications. By doing so, it seeks to enhance awareness and improve clinical practices surrounding this unique cutaneous tumor, ultimately promoting better patient outcomes in managing eyelid lesions.

## Case presentation

**Figure 1:**
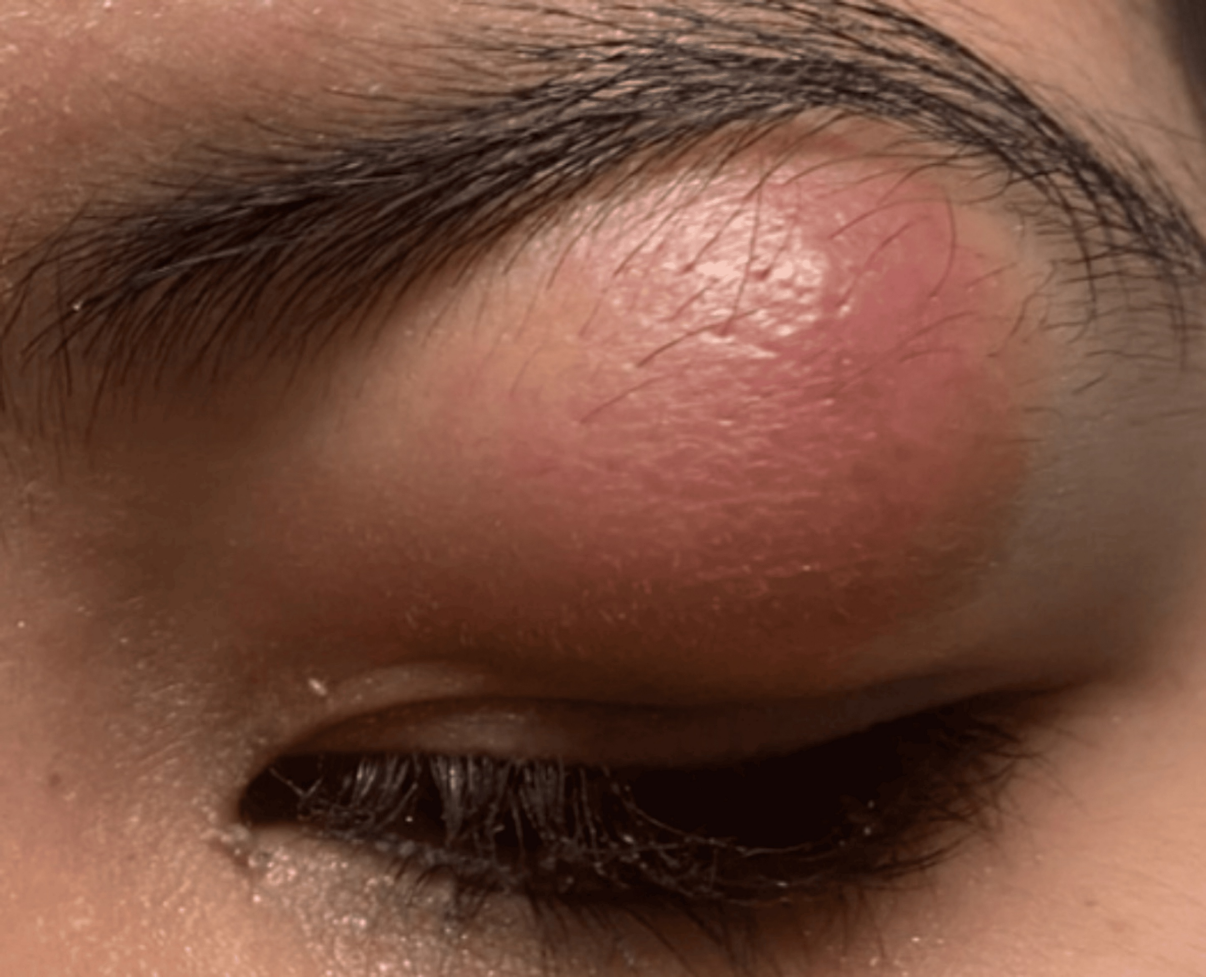
External photograph of the eyelid lesion

A 13-year-old female patient with a history of multiple lipomas on her back and arms but no other past medical history presented to the emergency department for a two-month history of left upper eyelid swelling. She had no family history of cancers or tumors. The patient had no other systemic abnormalities, and her labs were all normal. The lesion had grown rapidly in recent days, and her eyelid became red and painful. She had pain with supraduction but denied vision changes or diplopia. On exam, her visual acuity was 20/20 in both eyes, intraocular pressure was normal, and supraduction was limited in both eyes, likely secondary to pain. She had left upper eyelid ptosis and a large, well-circumscribed, subcutaneous mass extending from her eyebrow to the eyelid crease that was firm and tender to palpation. The external photograph of the lesion is shown in Figure 1.

**Figure 2:**
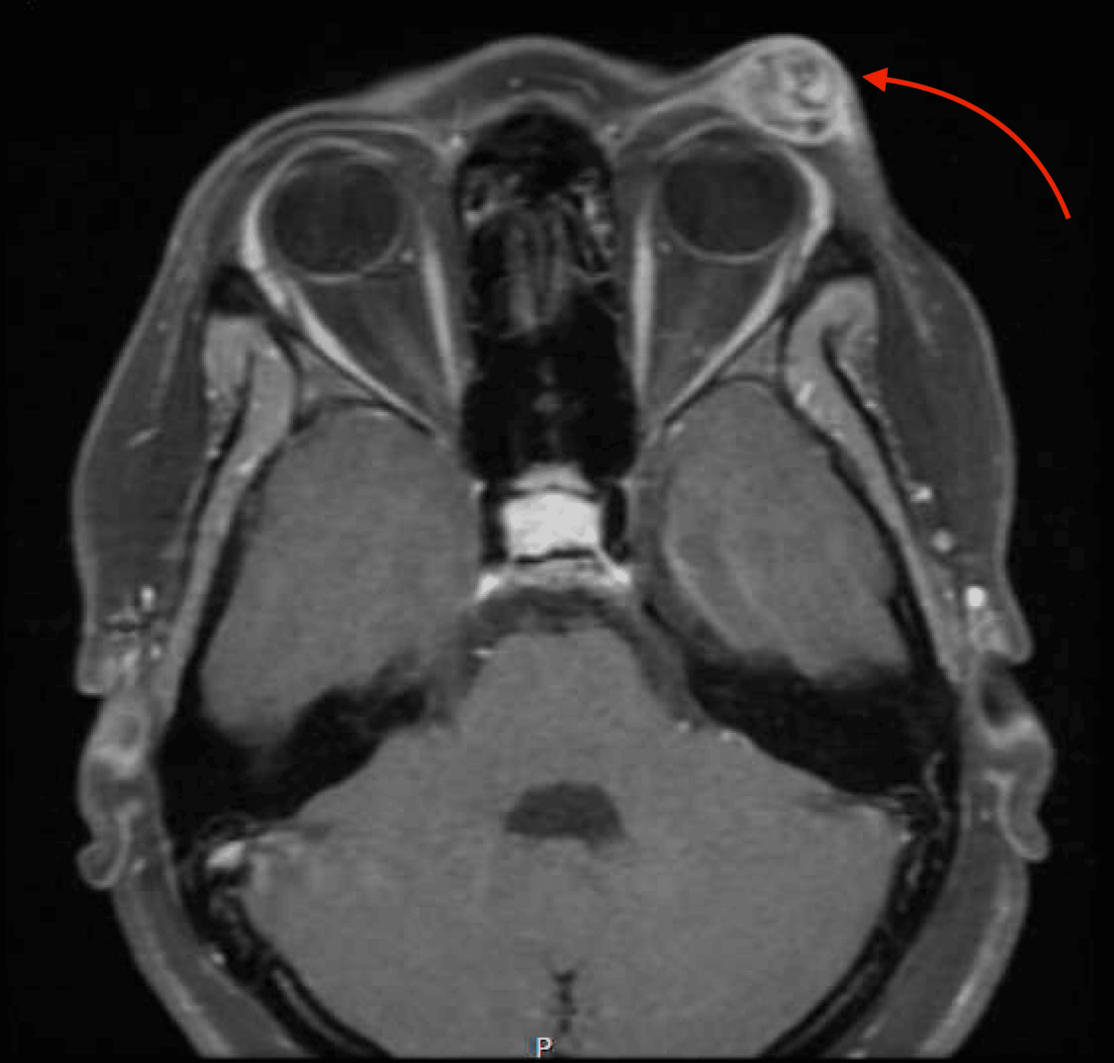
An axial T1 fat-saturated MRI of the orbit showing a large heterogeneous mass on the left eyelid MRI: magnetic resonance imaging

The patient's fundus exam was normal. Magnetic resonance imaging (MRI) of the brain and orbit with contrast showed a 2.5-cm enhancing upper eyelid tumor just anterior to the superior orbital rim, which appeared to originate from the frontalis muscle and was read as concerning for rhabdomyosarcoma (Figure 2).

**Figure 3:**
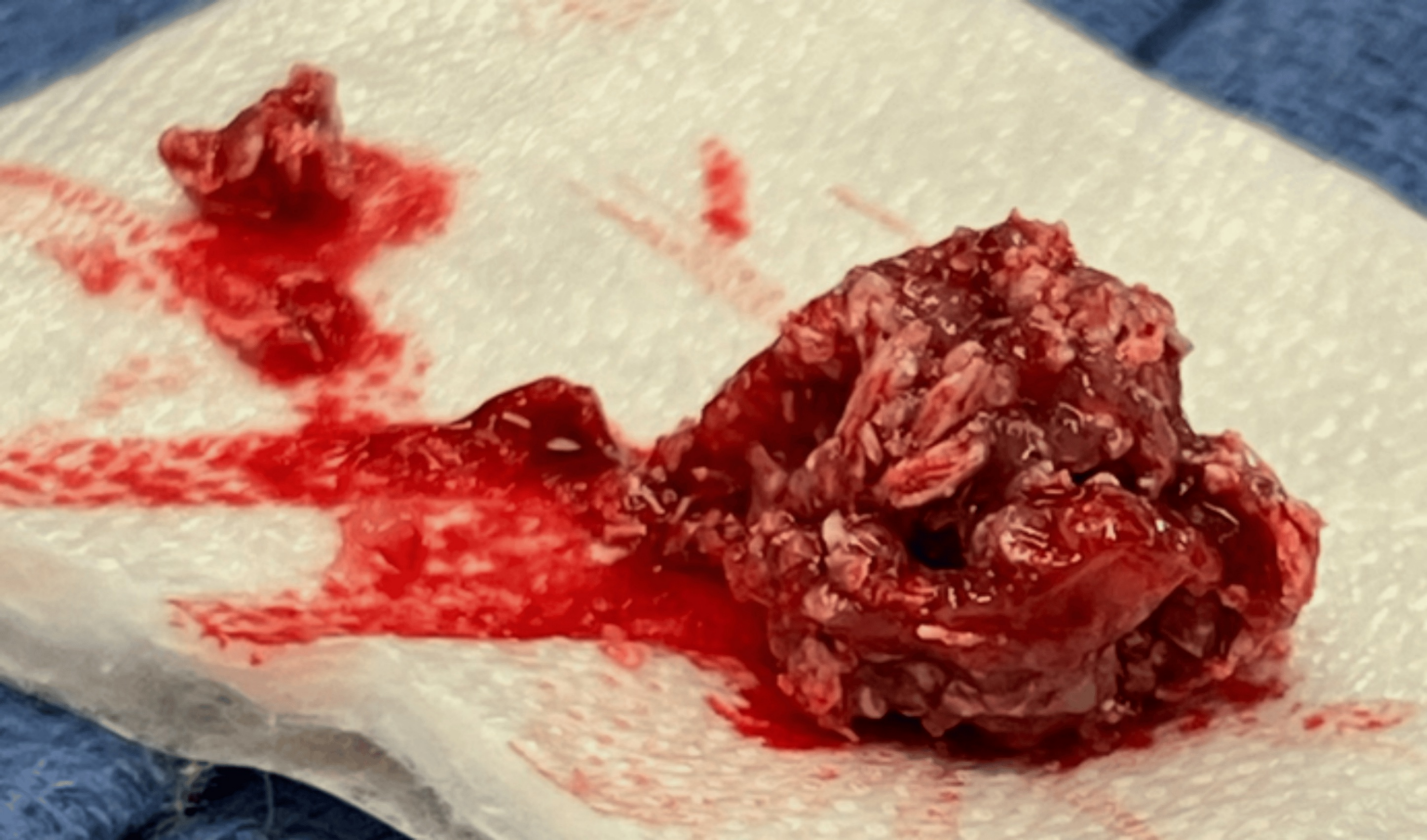
Gross specimen of the mass of the left upper eyelid

After discussing with oncology the patient was taken to the OR urgently for an incisional biopsy. During surgery, the mass was poorly adherent to the surrounding tissue and easily removed completely using gentle, blunt dissection. The gross specimen showed soft, white grains of calcification throughout the lesion (Figure 3).

**Figure 4:**
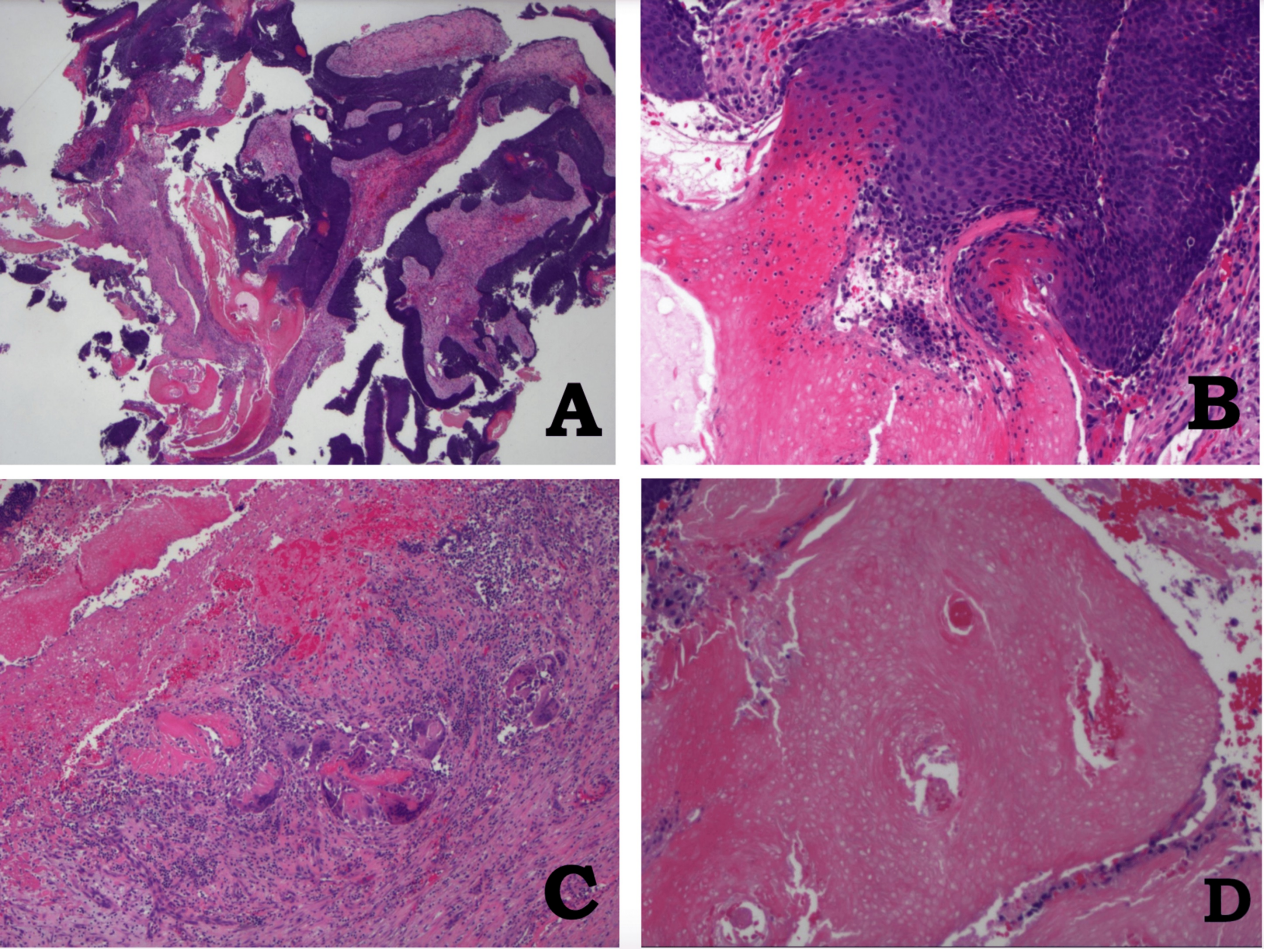
Pathology photographs of a lesion on the left upper eyelid. (A) Low magnification, panoramic view of the lesion, showing basaloid cells, eosinophilic cells (ghosts or shadow cells), and stromal fibrosis (H&E, 12.5×). (B) Basaloid cells (upper right) gradually or abruptly merged with keratinizing eosinophilic cells (also known as ghosts or shadow cells, lower left) (H&E, 100×). (C) Stroma with chronic granulomatous inflammation; foreign-body multinucleated giant cell reaction to keratin is seen (H&E, 40×). (D) Higher magnification of eosinophilic cells. The basaloid cells have become shadow/ghost cells, without nuclei (H&E, 100×) H&E: hematoxylin and eosin

Histopathological analysis revealed basaloid areas, focal calcifications, inflammation with foreign body giant cell reactions, and shadow (ghost) cells, consistent with pilomatrixoma (Figure 4).

After the resection, erythromycin ointment was prescribed four times a day over the lesion for 10 days. The incision site healed well with no complications. The patient did not require further treatment, and no recurrence was identified after six months.

## Discussion

Methods

A comprehensive literature search was conducted using electronic databases, including PubMed, Google Scholar, Ovid, and the Loma Linda electronic library. The search terms included "pilomatrixoma", "eyelid pilomatrixoma", and "eyelid tumors". The search was limited to articles published in the English language or formally translated into English and focused primarily on studies from the last two decades (2000-2024) to ensure current relevance. However, four studies were included from the late 1900s. Thirty-eight articles regarding pilomatrixomas of the eyelid were reviewed. Two articles were excluded from the study because we could not access the full articles, which included patient demographic information. Studies that did not clearly specify the location of the pilomatrixoma as being in the eyelid were excluded. Relevant data were extracted from the selected articles, including authorship, year of publication, study design, sample size, demographic information of patients, clinical characteristics, diagnostic modalities, treatment approaches, and reported outcomes. These data were systematically organized to facilitate comparative analysis.

Literature review

**Figure 5:**
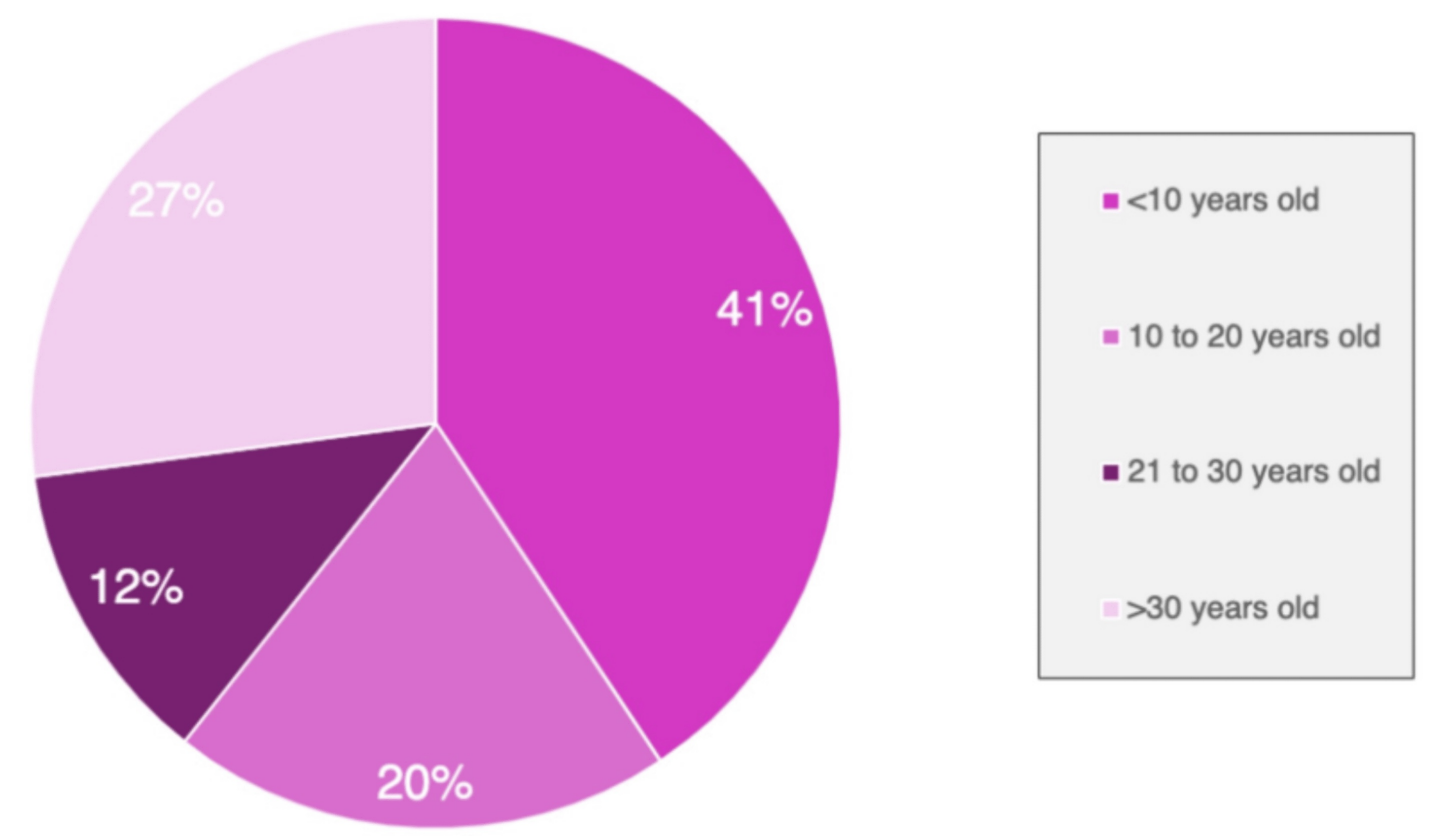
Age of patients with pilomatrixoma in percentiles recorded in the literature

In this study, 36 case reports [2,4,9,11,13-36], series [37-39], and retrospective studies [1,5,40-43] were reviewed, which include a total of 232 patients diagnosed with pilomatrixoma of the eyelid. These studies included 28 case reports, two case series, and six retrospective studies. They most commonly occur on the head and neck, with only about 11%-17% occurring in the periorbital region [12,15]. They occurred most commonly in the first decade of life but can occur at any age (Figure 5).

**Figure 6:**
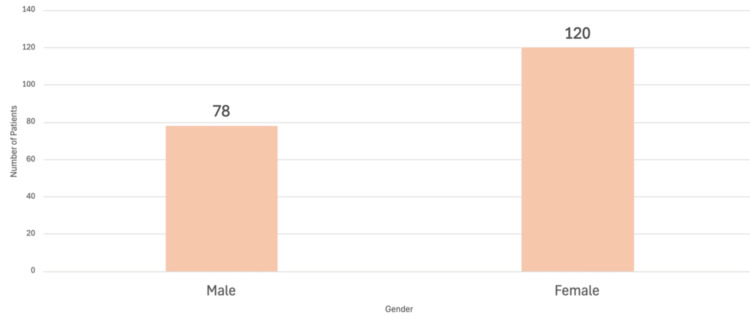
Gender of patients with eyelid pilomatrixomas recorded in the literature

Pilomatrixomas were more likely to occur in women (120/198) than in men (78/198). Not every author recorded gender; it was only recorded in 198 cases. The gender distributions of the recorded patients with pilomatrixoma are shown in Figure 6.

**Figure 7:**
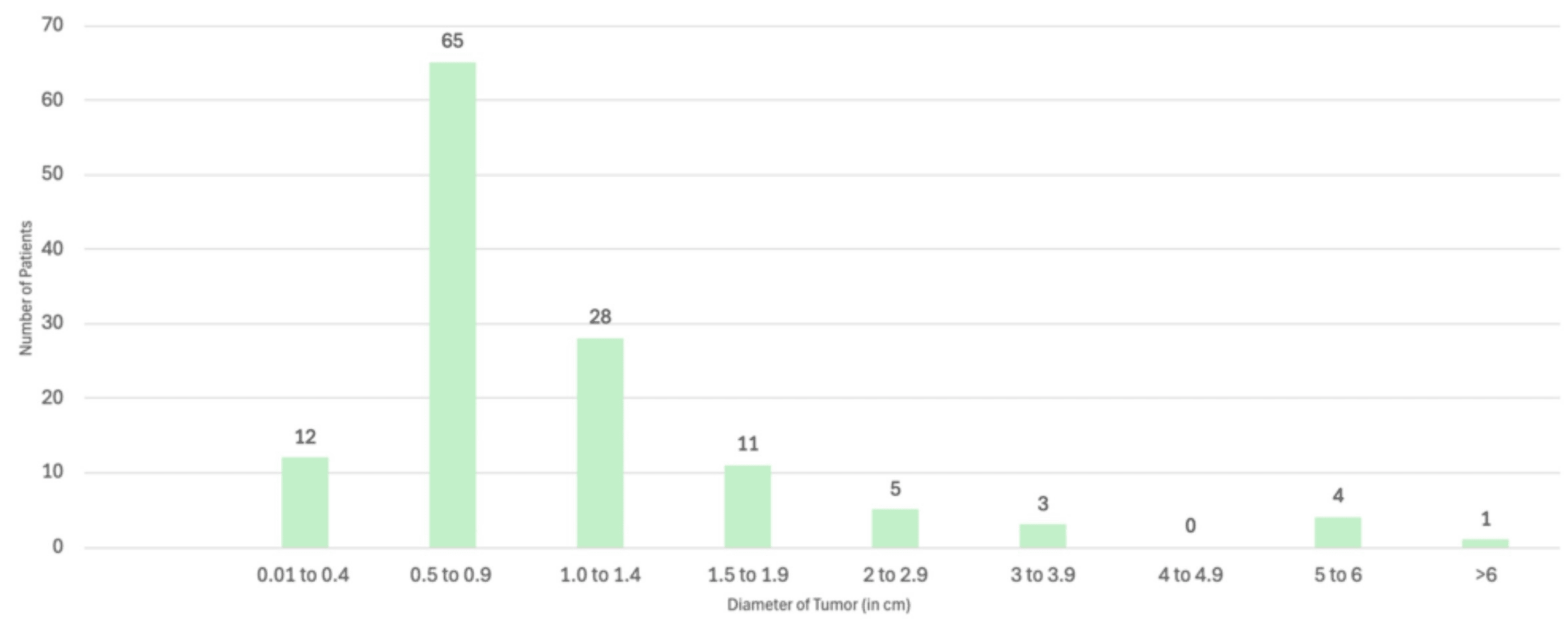
Graph showing the sizes of eyelid pilomatrixomas using the largest diameter of the tumor recorded in the literature

Most tumors were 5-10 mm measured as the largest diameter, and only 13 of the tumors were found to be greater than or equal to 2 centimeters in diameter. Rajabi et al. recorded the largest tumor as 7 cm × 10 cm [16]. A graph of the largest diameter of the pilomatrixoma reported in the literature is shown in Figure 7. One hundred twenty-nine patients in the literature had measurements of the tumor recorded.

A common theme among different authors was the difficulty of correctly diagnosing pilomatrixoma clinically. In all the papers that discussed a preoperative differential diagnosis, only 17.1% of the time did the initial evaluating clinician include pilomatrixoma in the differential or presumptive diagnosis before obtaining pathology results.

The most common location for the periorbital tumor was the upper eyelid or eyebrow, with 73%-81% of the cases of pilomatrixoma reported in the literature found on the upper eyelid or brow [40,43]. This is likely due to the presence of more hair follicles in that area [5].

Interpretation

Pilomatrixomas are rare, benign tumors of the hair matrix cells, with an estimated prevalence of 1% of all benign skin lesions [1]. There are currently no published guidelines on the workup of a pilomatrixoma. The clinical case described above is a great example of the typical presentation of an eyelid pilomatrixoma. It occurred in a female, which is the more common gender for presentation. It occurred in her second decade of life, which is slightly older than the average presentation. It occurred right below her eyebrow, which is the most common location for eyelid pilomatrixomas. The most unusual aspect of the above presentation is the size of the lesion, which was much larger than the average.

The eyelid lesion described above was initially concerning for a different diagnosis, which is quite common for this lesion. The difficulty in diagnosis occurs for several reasons. First, it is infrequently suspected due to the rarity of the lesion. Second, a pilomatrixoma can masquerade as several other more common lesions, including chalazions, cysts, and other tumors.

Clinical findings may result in a diagnosis, particularly for a classic presentation in a typical location. However, the clinical picture is not always enough. Other modalities utilized when the clinical picture is not straightforward include US, fine needle aspiration (FNA), CT, positron-emission tomography, MRI, and biopsy. These methods have variable efficacy rates in diagnosing a pilomatrixoma. According to Lim et al., US resulted in a diagnosis 76% of the time [12]. Generally, on US, the pilomatrixoma is described as an ovoid, well-defined, heterogeneous, hyperechoic subcutaneous mass with or without posterior shadowing [12]. Typical CT imaging findings of pilomatrixoma will show a well-circumscribed mass with calcifications present [7,12]. Han et al. reported that FNA with cytology resulted in a diagnosis 71% of the time. However, this modality commonly misdiagnosed findings for carcinoma [44]. Frequently, the diagnosis relies on biopsy. Siadati et al. describe the pathological features of eyelid pilomatrixoma. Pathology will show a well-circumscribed multilobular lesion, occasionally with a pseudocapsule. The lesion is biphasic. In the periphery, there are basophilic matrical cells with large nuclei. More centrally, the matrical cells lose their nuclei, and the cytoplasm becomes eosinophilic. At this point, the matrical cells have transitioned into “shadow cells,” which are a key feature of pilomatrixomas [1]. The body mounts a reaction to these shadow cells, often resulting in giant cells within the lesion. Calcification and ossification are also common [5]. The differentiation between aggressive pilomatrixomas and the malignant form, pilomatrix carcinoma, can be difficult; however, pathology typically shows greater anaplasia and an infiltrating border [45,46].

The treatment for the vast majority of cases in this review was complete excision. Recurrence rates after excision are low at approximately 2% [47]. According to Jones et al.’s review of pilomatrixomas, recurrence can occur any time from months to decades later [48]. Thus, actual recurrence rate may be higher due to inadequate follow-up period. Rarely, recurrence may be a sign of malignant transformation [47].

Due to the rarity of this tumor, no large retrospective studies have been published to date, with the largest study including only 77 patients [40]. Only one other study had a sample size larger than 20 [41]. Limitations to this review include potential publication bias due to only the unusual presentations of eyelid pilomatrixomas being published and inherent variability in reporting across different studies.

## Conclusions

Pilomatrixomas are a rare cause of eyelid lesions. They commonly occur within the first decade of life and are more common in women. They are typically small, well-circumscribed, slow-growing masses found most commonly on the upper eyelid or brow. Pilomatrixomas of the eyelid are difficult to diagnose, particularly when the presentation is atypical, and the diagnosis of pilomatrixoma is often overlooked due to its rarity. It is important to consider pilomatrixoma in the differential diagnosis of eyelid lesions and masses. Treatment consists of complete surgical excision. Although uncommon, recurrence is the most common complication. Future research might include further investigating risk factors, pathogenesis, and more long-term analysis of recurrence rates following excision.
